# Electromagnetic field tapering using all-dielectric gradient index materials

**DOI:** 10.1038/srep30661

**Published:** 2016-07-28

**Authors:** Jianjia Yi, Gérard-Pascal Piau, André de Lustrac, Shah Nawaz Burokur

**Affiliations:** 1 State Key Laboratory of Integrated Services Networks, Xidian University, 710071 Xi’an, Shaanxi, China; 2 Centre de Nanosciences et de Nanotechnologies, CNRS, Univ Paris Sud, Université Paris-Saclay, C2N–Orsay, 91405 Orsay cedex, France; 3 AIRBUS Group Innovations, 92150 Suresnes, France; 4 Université Paris Ouest, 92410 Ville d’Avray, France; 5 LEME, EA 4416, Université Paris Ouest, 92410 Ville d’Avray, France

## Abstract

The concept of transformation optics (TO) is applied to control the flow of electromagnetic fields between two sections of different dimensions through a tapering device. The broadband performance of the field taper is numerically and experimentally validated. The taper device presents a graded permittivity profile and is fabricated through three-dimensional (3D) polyjet printing technology using low-cost all-dielectric materials. Calculated and measured near-field mappings are presented in order to validate the proposed taper. A good qualitative agreement is obtained between full-wave simulations and experimental tests. Such all-dielectric taper paves the way to novel types of microwave devices that can be easily fabricated through low-cost additive manufacturing processes.

Transformation Optics (TO) allows controlling light in unprecedented and unbelievable ways through the use of judiciously engineered materials with electromagnetic material parameters that vary spatially^1^,^2^. Such flexibility in controlling the path of light appears to be convenient in the design of novel devices with performances or special properties difficult to achieve and has therefore inspired considerable research interests in the field of light propagation. The best known design conceived by this approach is the electromagnetic cloak in the microwave regime^3^, which is able to hide a space sector with regard to the incident light. Afterwards, TO concept combined with metamaterial engineering technology has resulted in the development of other conceptual and functional devices in the field of waveguiding^4^,^5^,^6^,^7^,^8^,^9^, illusion optics^10^,^11^,^12^,^13^,^14^ devices and antennas^15^,^16^,^17^,^18^.

As generally observed in TO-based devices, the designed structures exhibit anisotropy and spatial inhomogeneity and sometimes present a challenge for practical implementations, leaving lots of devices studied theoretically and unrealized experimentally. The resonant nature of constituting structures of TO-based devices, such as split ring resonators (SRR)^19^ and electric LC (ELC)^20^ resonators, limits the frequency bandwidth and functionality performances of the devices. Quasi-conformal transformation optics (QCTO) technique allowing the design of devices with quasi-isotropic dielectric materials has therefore been proposed^21^. QCTO has then been used to design quasi-isotropic devices for cloaking^21^, focusing^22^,^23^,^24^,^25^, magnification^26^, and beam steering applications^27^. The particularity of QCTO concept is that it allows minimizing the anisotropy of the constitutive materials, hence giving the possibility to implement devices from solely non-resonant dielectric materials. As such, nearly-isotropic graded index (GRIN) materials with broadband characteristics can be utilized, paving the way to broadband performances in functional devices.

Here, we use QCTO to design a coupling device capable of tapering the electromagnetic fields between two sections of different dimensions. We use the Laplace’s equation to determine the transformation medium. The electromagnetic waves exiting from a large section or aperture are properly guided through the taper to a smaller one. Full wave simulations based on finite element method is used to validate the proposed design and an all-dielectric tapering device is fabricated through 3D printing technology. Measurements are performed and reported experimental near-field mappings demonstrate a guiding of electromagnetic waves over a wide frequency range.

## Results

### Design of the field tapering device

**Figure 1:**
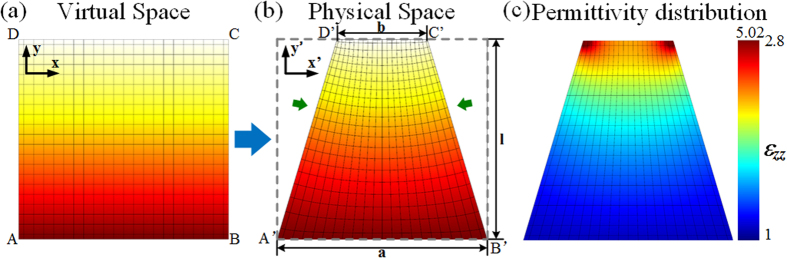
Illustration showing the space mapping from the virtual space to the physical space where the mesh grid represents the contour lines of *x* and *y* from the virtual space. (**a**) Air-filled initial virtual space. (**b**) Calculated transformed taper. (**c**) Calculated simplified permittivity (*ε*_zz_) distribution. *ε*_zz_ varies from 1 to 5.02 throughout the taper.

The experiment aiming to tailor the path of electromagnetic waves starts with the design of the taper. The virtual and physical spaces are respectively denoted by (*x,y*) and (*x’,y’*). The idea behind the transformation is to compress a part of the space in which the waves propagate. The air-filled rectangle *ABCD* in the virtual space is compressed into a quadrilateral *A’B’C’D’* in the real physical space, as illustrated by the schematic principle in Fig. 1. The points *A’* and *B’* in the physical space share the same location as *A* and *B* in the virtual space. We consider the length of the wide input segment *A’B’* to be equal to *a* and that of the narrow output *C’D’* to be *b*. The length of the taper is taken to be *l*.

The design is based on quasi-conformal transformation optics (QCTO) and achieved by solving Laplace’s equation. Neumann and Dirichlet boundary conditions are set at the edges of the taper:


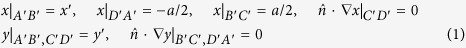


The solution allows avoiding the use of resonant metamaterials. Therefore the device can potentially present a large operational frequency bandwidth. Since Maxwell’s equations do not change form under coordinate transformations, the material parameters obtained after choosing a proper polarization are


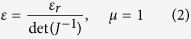


where 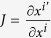


The permittivity (*ε*_zz_) distribution ranges from 1 to 5.02 for the calculated taper, supporting isotropic material parameters. It starts from 1 at the edge of the input and increases to 5.02, which is mostly distributed at the edges of the output region, as shown in Fig. 1c.

### Implementation of the taper

**Figure 2:**
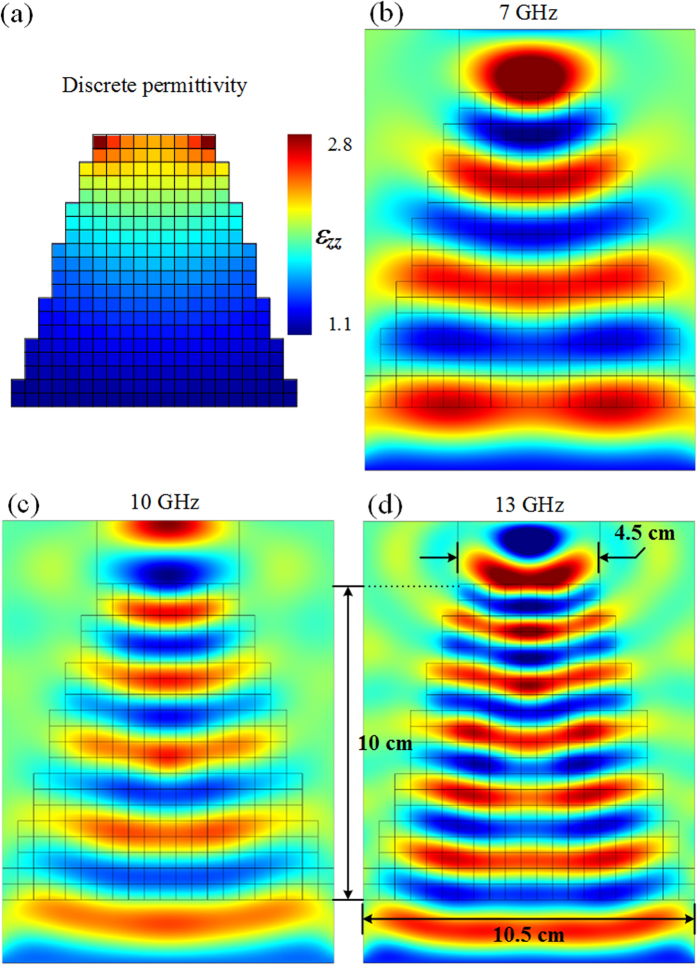
Discretization of the field tapering device. (**a**) Calculated discrete taper with *ε*_zz_ varying from 1 to 2.8. 2D simulated electric field distributions of discrete field tapering device at (**b**) 7 GHz, (**c**) 10 GHz, (**d**) 13 GHz.

Using QCTO, the coupler design can be implemented using isotropic all-dielectric materials relying only on gradients in the index of refraction of the medium. The taper material parameters are therefore non-magnetic and inhomogeneous. We fabricate the taper using additive manufacturing process. 3D polyjet printing is utilized so as to consider air holes of different geometrical dimensions in a dielectric host medium to obtain the gradient distribution of the index of refraction. The discrete dielectric permittivity distribution of the printed taper is depicted in Fig. 2a. The discrete taper model is composed of 300 unit cells, with dimensions of 5 mm × 5 mm each. The respective permittivity of each cell is considered to be constant across the cell and is equal to the average permittivity within the cell. As illustrated by the color plot in Fig. 2a, the permittivity *ε*_*zz*_ values range from 1 to 2.8 in the discrete approximation.

Finite element method based numerical simulations are used to design and characterize the proposed transformed field tapering device. 2D modeling is performed by the use of the commercial software Comsol Multiphysics^28^. Scattering boundary conditions are set around the computational domain, and a line source is applied to the input boundary (segment *A’B’*). The length of the segments *A’B’* and *C’D’* is respectively set to *a* = 10.5 cm and *b* = 4.5 cm. The total length of the taper *l* = 10 cm. The isotropic effective property obtained from Laplace’s equation is assigned to the taper region.

The simulated electric field distribution is calculated at 7 GHz, 10 GHz and 13 GHz respectively. As it can be clearly observed, the taper compresses the emitted beam waist from 10.5 cm wide to 4.5 cm, with almost no leakage outside the taper region. The intensity of the field increases along the direction of the wave propagation, which means a field concentration and therefore a promising field coupling effect. The taper also shows wideband performances from 7 GHz to 13 GHz.

**Figure 3:**
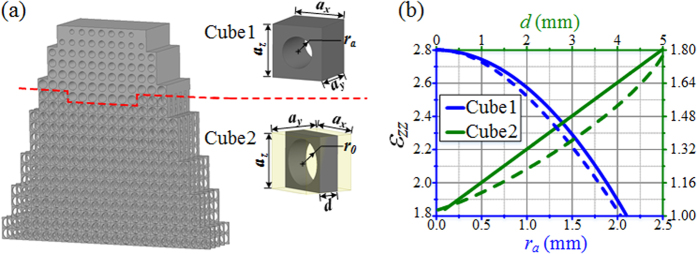
(**a**) Design of 3D discrete taper composed of two different kinds of unit cells. (**b**) Effective permittivity of the cell composed of an air hole in a dielectric host medium. A parametric analysis is performed to extract the effective permittivity value according to the radius *r*_*a*_ of the air hole for cube 1 (with *d* equal to *a*_*y*_) and to the thickness *d* for cube 2. The continuous traces correspond to approximate values calculated using the mixing formula and the dashed traces correspond to accurate values retrieved from homogenization procedure.

Figure 3a shows the design of a realistic tapering device made of a dielectric material diluted with air holes. We design the meta-taper to operate in a broad frequency range spanning from 7 to 13 GHz. Two kinds of unit cells are considered as metamaterial building blocks to realize the required permittivity distribution. The size of each unit cell is taken to be *a*_*x*_ × *a*_y_ × *a*_z_ mm^3^, as presented in Fig. 3a. Cubic cells holding the two kinds of structures are used to supply the particular values of permittivity, where *a*_*x*_ = *a*_y_ = *a*_z_ = 5 mm and *r*_0_ = 2.1 mm.

By adjusting the volume fraction of the air holes in the dielectric host medium, either by varying the radius *r*_a_ of the air hole or by adjusting the thickness *d* of the hole, the effective permittivity of the cell can then be modified, as shown in Fig. 3b. Mixing formula has in a first step been used to approximate the effective permittivity of the unit cells. When we consider two materials mixed together, the effective parameter can be approximated by:


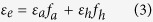


where *ε*_a_ = 1 and *f*_a_ and *f*_h_ are the volume fraction of the air holes and the host material, respectively. Reflection and transmission coefficients of a unit cell have afterwards been utilized to retrieve the accurate material parameters^29^.

**Figure 4:**
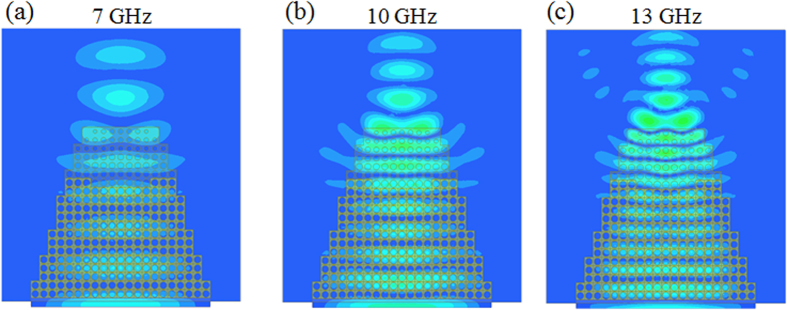
Simulated electric field distributions of 3D taper in the *x-y* plane at (**a**) 7 GHz, (**b**) 10 GHz and (**c**) 13 GHz.

Full-wave simulations using Ansys HFSS^30^ have been performed on the 3D discrete device to verify the field tapering functionality. The 3D taper model consists of 300 cubic cells containing two different types of building blocks. A wave port is used as emitting source at 7 GHz, 10 GHz and 13 GHz as presented in Fig. 4. As it can be observed, in presence of the designed 3D dielectric taper, the beam waist of the radiated electric field is reduced along the shape of the taper, confirming the 2D simulation results and the fact that the device is able to taper the field.

### Experimental characterization of the all-dielectric taper

**Figure 5:**
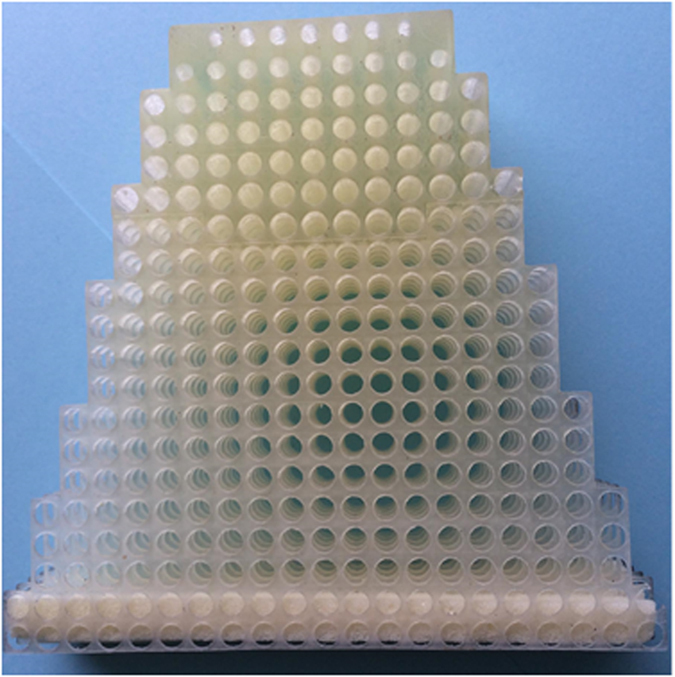
Photography of the fabricated all-dielectric taper prototype.

The prototype is printed using a dielectric material of relative permittivity *ε*_h_ = 2.8 and consists of 300 unit cells. A photography of the fabricated taper prototype is presented in Fig. 5. To validate the tapering functionality, an experimental system aiming to scan the electric near-field microwave radiation is set up. The electric field is scanned by a field-sensing monopole probe connected to one port of a vector network analyzer by a coaxial cable. The probe is mounted on the lower plate of a two parallel plate waveguide, while the taper is fixed on the upper plate between two parallel plates. The probe on the lower plate mounted on computer-controlled translation stages can be moved within the radiation region of the system under test. The field sensor in stepped in increments of 2 mm and the field amplitude and phase are recorded at every step. A full 2D spatial field distribution of the microwave near-field pattern is then mapped in free-space. The total scanning area can cover a surface area of 150 × 160 mm^2^. A broadband horn antenna is used as wave launcher at the wide input of the taper. Microwave absorbers are applied around the measurement stage in order to suppress undesired scattered radiations.

**Figure 6:**
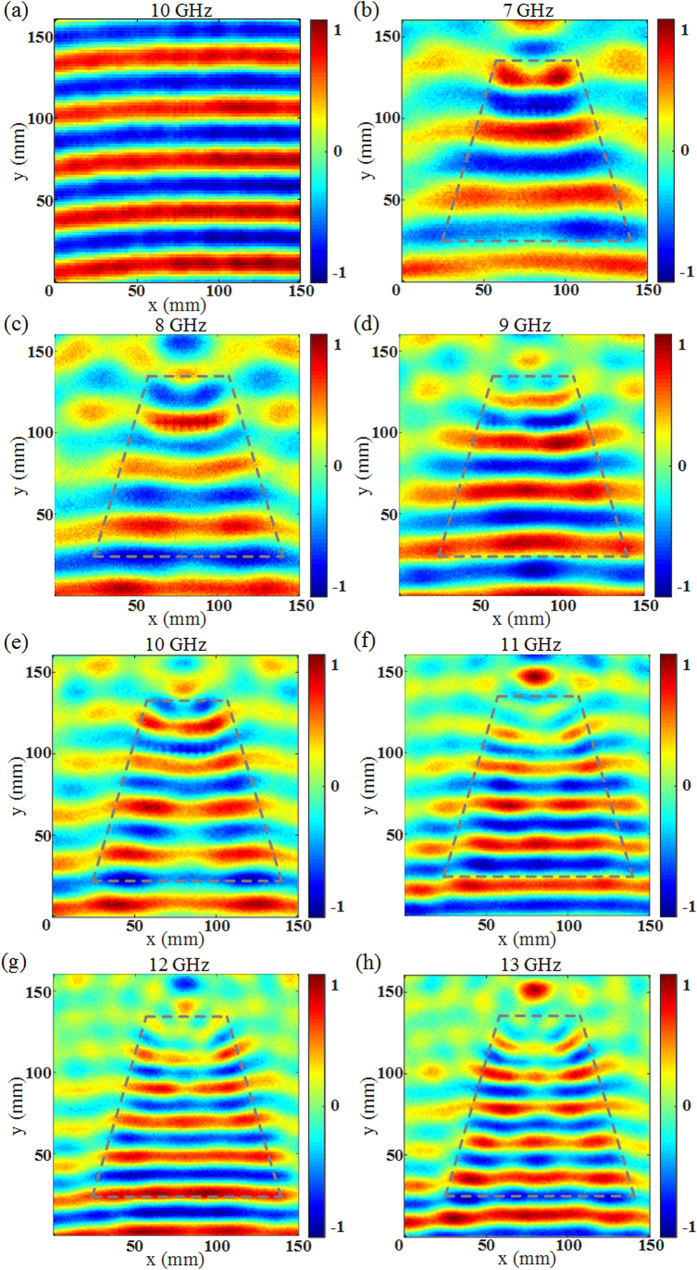
Normalized measured electric near-field distribution. (**a**) Free space at 10 GHz. (**b**–**h**) Top surface of the taper from 7 GHz to 13 GHz.

To experimentally validate the proposed QCTO-based taper, the fabricated prototype having dimensions *a* = 10.5 cm, *b* = 4.5 cm and *l* = 10 cm, is measured in a wide frequency range from 7 to 13 GHz. Figure 6a shows the field wavefronts in free space as a reference. The electric field cartography is scanned along the top surface of the taper and is depicted in Fig. 6b–h for 7, 8, 9, 10, 11, 12 and 13 GHz. In all figures, the wide waist of the field wavefronts is narrowed when propagating through the taper. The coupling of the field from a wide section to a narrower one can be evidently observed. The broadband performance and the low-cost realization make the device very attractive.

## Discussion

In summary, we have presented the theoretical design and experimental realization of a compact all-dielectric field tapering device operating in microwave regime on a wide frequency range. The all-dielectric taper has been tested over a broad frequency band spanning from 7 GHz to 13 GHz. A horn antenna is used to send field wavefronts at the input of the taper. Such a taper is able to gradually reduce the beam waist of the field wavefronts. The concept has been validated through calculated and measured near-field distributions. The proposed method is ease of fabrication, low-cost and presents potential applications in microwave devices.

Compared to conventional method using several quarter-wavelength tapers for a smooth impedance matching, the proposed transformed taper allows to transmit electromagnetic waves by compressing the field in a tapered profile. The device can operate both between closed waveguides, and in free space on a broad frequency band. It can also be much shorter than the classical system of multiple quarter-wavelength tapers when higher gradient in permittivity is applied.

Transmission losses in the proposed taper are due to the dielectric losses and also to the difference in the refractive index at the boundary of the output of the taper device with vacuum. A suitable matching zone at the output of the taper will allow to decrease the losses in an efficient manner.

**Figure 7:**
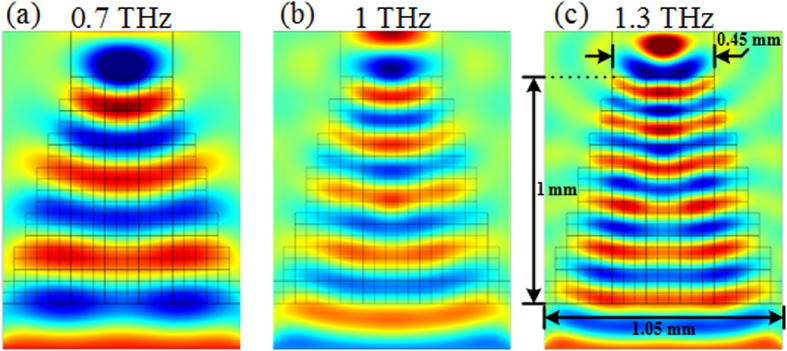
Simulations performed at THz frequencies, showing 2D simulated electric field distributions of the taper at (**a**) 0.7 THz, (**b**) 1 THz, and (**c**) 1.3 THz.

The concept can be easily extended to the THz or higher frequency regimes simply by downscaling the physical dimensions of the device. Electric field distributions from simulations performed on a 2D model having dimensions *a* = 1.05 mm, *b* = 0.45 mm and *l* = 1 mm are presented in Fig. 7. It can be clearly observed that similar field tapering can be obtained as at microwave frequencies.

## Methods

### Fabrication of the taper

The taper is fabricated using the Objet Eden260VS 3D printer^31^. The 3D printing is based on the polyjet technology consisting in jetting layers of curable liquid photopolymer onto a build tray. During the printing process, the photopolymer is jetted and is instantly cured by ultra-violet (UV) light. Fine layers of the UV-cured polymer are accumulated onto a build tray. The air holes are filled with a gel-like material that is easily removed with water.

## Additional Information

**How to cite this article**: Yi, J. *et al*. Electromagnetic field tapering using all-dielectric gradient index materials. *Sci. Rep.* **6**, 30661; doi: 10.1038/srep30661 (2016).
